# Crosstalk between Tumor-Infiltrating Immune Cells and Cancer-Associated Fibroblasts in Tumor Growth and Immunosuppression of Breast Cancer

**DOI:** 10.1155/2021/8840066

**Published:** 2021-07-13

**Authors:** Jarupa Soongsathitanon, Pranisa Jamjuntra, Nuttavut Sumransub, Supaporn Yangngam, Marjorie De la Fuente, Glauben Landskron, Peti Thuwajit, Marcela A. Hermoso, Chanitra Thuwajit

**Affiliations:** ^1^Department of Immunology, Faculty of Medicine Siriraj Hospital, Mahidol University, Bangkok 10700, Thailand; ^2^Graduate Program in Immunology and Department of Immunology, Faculty of Medicine Siriraj Hospital, Mahidol University, Bangkok 10700, Thailand; ^3^Programa Disciplinario de Inmunología, Instituto de Ciencias Biomédicas, Facultad de Medicina, Universidad de Chile, Santiago, CL 8380453, Chile; ^4^Dirección Académica, Clínica Las Condes, Santiago, CL 7591018, Chile

## Abstract

Signals from the tumor microenvironment (TME) have a profound influence on the maintenance and progression of cancers. Chronic inflammation and the infiltration of immune cells in breast cancer (BC) have been strongly associated with early carcinogenic events and a switch to a more immunosuppressive response. Cancer-associated fibroblasts (CAFs) are the most abundant stromal component and can modulate tumor progression according to their secretomes. The immune cells including tumor-infiltrating lymphocytes (TILs) (cytotoxic T cells (CTLs), regulatory T cells (Tregs), and helper T cell (Th)), monocyte-infiltrating cells (MICs), myeloid-derived suppressor cells (MDSCs), mast cells (MCs), and natural killer cells (NKs) play an important part in the immunological balance, fluctuating TME between protumoral and antitumoral responses. In this review article, we have summarized the impact of these immunological players together with CAF secreted substances in driving BC progression. We explain the crosstalk of CAFs and tumor-infiltrating immune cells suppressing antitumor response in BC, proposing these cellular entities as predictive markers of poor prognosis. CAF-tumor-infiltrating immune cell interaction is suggested as an alternative therapeutic strategy to regulate the immunosuppressive microenvironment in BC.

## 1. Introduction

Breast cancer (BC) is the most frequent cancer among women worldwide representing a global health problem. It was estimated in 2018 that more than 2.1 million women were newly diagnosed with BC, with 600,000 deaths [1] and 2.3 million new cases are estimated by 2030 [2]. The survival rate of patients has improved in recent decades due to early diagnosis and better access to treatments, but it is still lower in developing regions [2]. Although early-stage and nonmetastatic BC is curable, advanced disease with distant organ metastasis is considered incurable with current therapies [3].

BC can be classified into five subtypes according to the expressions of estrogen receptor (ER), progesterone receptor (PR), and epidermal growth factor receptor 2 (HER2), acting as predictive factors and guiding therapy decision-making [3]. These subtypes are (1) luminal A-like HER2^−^ (ER^+^/PR^+^/HER2^−^/Ki67^−^), (2) luminal B-like HER2^−^ (ER^+^/PR^+^/HER2^−^/Ki67^+^), (3) luminal B-like HER2^+^ (ER^+^/PR^+^/HER2^+^/Ki67^+^), (4) HER2-enriched or nonluminal subtype (ER^−^/PR^−^/HER2^+^), and (5) triple-negative BC (TNBC) (ER^−^/PR^−^/HER2^−^). Most patients are luminal A-like subtype, accounting for 60-70% of total cases, whereas the TNBC subtype is less frequent with around 10-15% incidence [3]. Based on histological subtypes, invasive ductal carcinoma (IDC) and ductal carcinoma in situ (DCIS) are the most commonly studied, with IDC the most common invasive BC, having an incidence of around 72-80% of total BC cases, whereas DCIS is the most frequent subtype of noninvasive BC accounting for a quarter of all cases [4]. DCIS is not generally considered as a life-threatening disease, although it can increase the risk of tumor progression to IDC.

Physiologically, the mammary gland tissue dynamically changes throughout a women's life, that is, the remodeling that occurs during pregnancy, lactation, involution (a process triggered postweaning), and regression. The immune system of the normal breast shares similarities with other mucosal systems, such as the intestine or respiratory mucosa, and plays a crucial role in protection and maintenance of the normal glandular structure [5]. It is also witnessed that carcinogenesis develops spontaneous breast adenocarcinomas in immunodeficient mice compared to immunocompetent mice [6].

It is well accepted that immune cells infiltrating into the tumor microenvironment (TME) have opposing functions such as T cells (cytotoxic CTLs, T helper type 1 (Th1)), natural killer cells (NKs), B cells, and mononuclear cells (M1-classically activated macrophages and mature dendritic cells (DCs)) contributing to tumor eradication, whereas Th2, regulatory T cells (Tregs), myeloid-derived suppressor cells (MDSCs), and alternatively activated macrophages (M2) or tumor-associated macrophages (TAMs) have protumorigenic functions [7, 8]. The low immunogenicity of BC and immunosuppressive TME limit immunotherapy benefits targeting the adaptive immune system, such as checkpoint inhibitors [9]. To achieve successful immunotherapy in BC, the functions of cellular components in TME that determine tumor immune response evasion need to be fully understood. Among these components, cancer-associated fibroblasts (CAFs) and tumor-infiltrating immune cells are our focus in this review. CAFs are involved in BC initiation, proliferation, invasion, and metastasis, playing a critical role in metabolic TME reprogramming and therapy resistance [10–13], secreting growth factors, cytokines, and hormones, together with extracellular matrix (ECM) paracrine effects and mechanical stimuli determining cancer development. Additionally, microenvironmental events including angiogenesis, lymphangiogenesis, ECM remodeling, cancer-associated inflammation, and metabolism reprogramming have premalignancy potency through signaling pathway crosstalk among CAFs, cancer cells, and ECM [13–16].

In this review article, the role of tumor-infiltrating immune cells and CAFs in BC is examined, including basic knowledge on immune cells and CAF phenotypes. Additionally, we discuss CAF immunosuppressive effect contributing to immune escape of BC. We hope that understanding the interaction between tumor cells and TME components (CAFs and immune cells) will bring forth new patient management and targeting therapies within BC.

## 2. Tumor Microenvironment of Breast Cancer (BC)

In BC TME, a combination of heterogeneous cell types communicates with cancer cells, including tumor-infiltrating immune cells, adipocytes, pericytes/endothelial cells, CAFs, and noncellular components such as ECM, cytokines, and growth factors [17–19]. The impact of cells of the immune system and CAFs on cancer progression, drug, and immunotherapeutic responses is discussed.

### 2.1. Immune Cells in BC Tissues with Their Anti- or Protumoral Functions and Roles in Prognosis and Therapy Response

In normal breast epithelium, the immune cell population varies according to the reproductive state. In nulliparous mouse models, immature DCs and ROR*γ*T^+^-Th17 cells are predominant. During lactation, DCs acquire a tolerogenic phenotype with decreased T cell activation, extending during weaning by FOXP3^+^ Treg expansion. These activated and tolerogenic programs seem to assure protection from bacterial and self-antigens during epithelial cell death, occurring after lactation and involution [5].

During acute lobulitis, an increased infiltration of CD45^+^ leucocyte (including CD4^+^-T cells, CTLs, and CD19^+^/CD20^+^-B cells) is seen compared to normal tissue without DC or CD68^+^-monocyte/macrophage changes [20].

Diverse immune cells are recruited during tumor development and progression in the mammary gland [20]. In benign lesions, high monocyte/macrophage and DC infiltration is seen, whilst decreased CTLs and absence of B cells are associated with increased risk of BC [21, 22]. In *in situ* transition of carcinoma to invasive carcinoma, changes in conventional cell populations (decreased CTLs and increased Tregs) can promote an immunosuppressive environment with immune escape [23]. This process is characterized by the loss of immunostimulatory molecules (i.e., major histocompatibility complex (MHC) class I, transporter-associated antigen processing (TAP) subunit 1), increased expression of immunoinhibitory molecules (i.e., programmed death-ligand 1 (PD-L1) and human leukocyte antigen-G (HLA-G)), and altered apoptosis component expression (i.e., Fas and FasL) [5], leading to disease progression and treatment failure. We review in this section the main immune cell populations and their role in prognosis and therapy response.

#### 2.1.1. Tumor-Infiltrating Lymphocyte Subsets

Lymphocytes present in tumor tissues, namely, tumor-infiltrating lymphocytes (TILs), are highly heterogeneous playing a crucial role in host antigen-specific tumor immune response. TILs have pro- or antitumor properties depending on T cell subsets in distinct cancer tissues with antitumor subsets, mainly CTLs and Th1 cells, whereas Th2, Th17, and Treg exhibit opposing roles.

In breast TME, TILs are predominantly activated T lymphocytes (CD3^+^/CD56^−^, CD4^+^, or CD8^+^-T cells) [10], with their increase associated with good prognosis in TNBC patients [24–26] and chemotherapy response in BC patients [24, 27–29].

**Figure 1:**
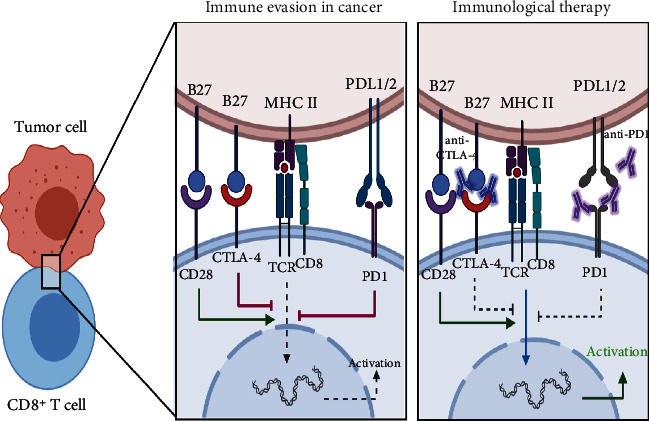
Targeting checkpoints of T cell in *Lum 3* breast cancer. TILs recognizing small peptide presented through MHC class I/II, with costimulatory signals necessary to modulate T cell activation. Indeed, the interaction between B7 and CD28 on TILs is a positive activation signal; however, in *Lum 3* BC, the CTLA-4 (CD28 competitive receptor) and PD1/PD-L1 pathway (immune and endothelial cells) delivers an inhibitory signal. Targeting immune checkpoints CTLA4 and PD1/PDL-1 can be used for different cancer treatments, including BC [31–35].

Using TIL gene signatures in luminal A and luminal B BC, predictive responses of immune checkpoint inhibitors, such as anti-CTLA4 and anti-PD1 (Figure 1), allow stratification into three subtypes: (1) *Lum 1*, with low TILs and immune gene levels, (2) *Lum 2*, high expression of *STAT1* and interferon-stimulated genes with TP53 somatic mutations, and (3) *Lum 3*, high level of TILs and immune-related/immune checkpoint gene expression (i.e., *PD-L1* and cytotoxic T lymphocyte-associated antigen 4 (*CTLA-4*)) and chemokine genes (i.e., *CXCL9* and *CXCL10*) and their receptors [30]. Lum 1 has low TILs whilst Lum 3 has high TILs with high immune checkpoint expressions, which may imply that Lum 3 may have better predictive response to immune checkpoint inhibitor (ICI), the drug that blocks immune checkpoint molecules resulting in restoration of the immune system function, than Lum 1. TILs recognize small peptide presented through MHC class I/II, with costimulatory signals necessary to modulate T cell activation. Indeed, the interaction between B7 and CD28 on TILs is a positive activation signal; however, in *Lum 3* BC, the CTLA-4 (CD28 competitive receptor) and PD1/PD-L1 pathway in immune cells and endothelial cells delivers an inhibitory signal (Figure 1). Targeting immune checkpoints CTLA-4 and PD1/PDL-1 can be used for different cancer treatments, including BC [31–35]. Effects of ICI or immune cell therapy are diminished by immunosuppressive TME status. Therefore, to enhance this immune therapy's efficacy in breast cancer, the combination with chemotherapy is a plausible option [36].

Another important antitumor cell is CTLs, inducing tumor cell lysis through perforin and granzyme release inducing cell apoptosis [37, 38]; however, during cancer progression, they become dysfunctional and exhausted due to TME immune-related tolerance and immunosuppression [39].

It is well accepted that high intratumor CTLs and memory TILs are associated with improved prognosis [38], as with Th1 cell in ER^−^ and TNBCs, which secrete several cytokines/chemokines, activating and recruiting CTLs, together with NK cells and M1 macrophages to eradicate breast tumor cells [40]. Thus, the presence of follicular Th-CXCL13^+^ cells can predict good survival and prognosis in BC patients [41, 42].

In opposition, Th2 mainly produces interleukin (IL-10), modulating the TME immunosuppressive profile to inhibit antigen-presenting cells (APCs) and effector cell function [40, 43]. Th17 is another subset promoting tumor growth and angiogenesis [44, 45], although whilst priming CTLs with antitumor activity and favorable prognosis [46–49], their role in cancer remains controversial [50]. Infiltration of IL-17/IL-17A-secreting cells is associated with poor prognosis in BC patients [51–53], enhancing tumor migration, invasion, and chemotherapy resistance [54, 55]; the IL-17B/IL-17 B receptor (IL-17RB) axis is associated with poor prognosis and chemoresistance [56, 57], through the NF-*κ*B/Bcl-2 antiapoptotic signaling pathway [58], becoming an attractive therapeutic target [59].

Tregs represent a minor CD4^+^-T cell population, maintaining immune homeostasis by inhibiting effector T cells [60], being increased in nearly all cancers associating with metastasis, tumor recurrence, and treatment resistance [61]. FOXP3^+^ is an indicator of Treg activity, tumor progression, and metastasis [62], with its suppression, together with programmed death 1 (PD1), T cell immunoglobulin mucin-3, and CTLA-4, assuring the increased immunotherapy response [61] (Figure 1), relapse-free survival (RFS), and overall survival (OS) in BC patients [63]. Treg-enriched TME potentiates immunosuppressive cells, i.e., CAFs, cancer cells, TAMs and MDSCs, whilst suppressing immunostimulatory cells, i.e., CTLs and NKs [61]. Nonetheless, Th2-derived IL-4 mediates Treg conversion to Th9 subset [64] with antitumor function increasing DC survival [65]. Finally, tumor-infiltrating B lymphocytes regulate cancer progression via IL-10 production, although no consensus at present exists on their benefit as a prognosis marker [66].

#### 2.1.2. Mononuclear Infiltrating Cells

Mononuclear myeloid cells are a heterogeneous population of bone marrow-derived cells which include monocytes, terminally differentiated macrophages, and DCs. Myeloid cells promote cancer progression by either directly interacting with tumor cells or supporting a tumor stroma that promotes tumor growth, angiogenesis, migration, invasion, and metastasis [67] and additionally suppressing tumor immunity [68]. The combined expression of high IL-1*β* and IL-6, but low IL-10 in stromal mononuclear inflammatory cells, associates with good prognosis and long relapse-free survival in breast carcinoma patients [69].

TAMs are a major constituent of TME in BC, mostly displaying the M2 phenotype with immunosuppressive activity, directly correlating with poor prognosis, activating cancer stem cells, cancer cell invasion, and tumor angiogenesis, and suppressing antitumor CTL functions [70].

Another important cell present in BC is DCs, classified into two major subsets: conventional DCs (cDCs, also known as myeloid DCs or classical DCs (cDCs)) and plasmacytoid DCs (pDCs) producing type I interferon (IFN) in response to nucleic acids [71]. As the conventional type 1 DC (cDC1) subset is superior in antigen cross-presentation (a process of exogenous antigen presentation on MHC class I), cDCs control tumor progression, resulting in CTL priming and activation. Moreover, the cDC1 subset is important in the immune control of tumor by enhancing local cytotoxic T cell function, making them significant in immune checkpoint blocking therapy [72, 73]. In TNBC, cDCs expressing activation marker CD11c^+^ directly correlate with TILs, CD4^+^, and CD8^+^ T cell counts, highlighting the potential therapeutic options to modulate their recruitment and function [74]. DCs from human primary luminal and TNBC tissues are enriched in vascular wound healing/ECM pathways and immunological IFN pathways, respectively. Additionally, the type of tumor impacts on the diversity of DC subset correlating with disease outcome [75], therefore providing potential targets and biomarkers to evaluate immune status of BC TME.

Unlike cDCs, pDCs migrate to lymphoid organs and peripheral blood upon development. Infiltration of pDCs into cancer tissues is associated with poor prognosis correlating with BC lymph node metastasis, with participation of the CXCR4/CXCL12 chemokine axis or stromal cell-derived factor 1 alpha (SDF-1*α*) [76]. High CXCL12/SDF-1 level is seen in metastatic lymph nodes and CXCR4 upregulation in cancer cell lines exposed to pDC conditioned media. Finally, the immunosuppressive TME alters DC differentiation into tolerogenic regulatory DCs [77], characterized by decreased DC maturation marker content and promoting Treg expansion [78–80].

#### 2.1.3. Myeloid-Derived Suppressor Cells (MDSCs)

MDSCs are a heterogeneous population of myeloid cells, contributing to an immune suppressive/anergic and tumor permissive environment [81] through inhibitory cytokines and other substances [82], thus promoting cancer progression and metastasis [81, 83], although there is no consensus for MDSC expression markers in tumor [84, 85]. MDSCs are usually absent in healthy individuals, appearing only in cancer and pathological conditions associated with chronic inflammation or stress [86]. This occurs in surgical removal of primary tumor in a xenograft mammary cell carcinoma murine model, demonstrating that MDSC infiltration is crucial to promote lung metastasis [87], by secreting transforming growth factor-*β*1 (TGF-*β*1), vascular endothelial growth factor (VEGF), and IL-10 inducing epithelial-mesenchymal transition (EMT). Thus, biological MDSC depletion therapy is a promising treatment strategy to prevent immune evasion after mastectomy in BC. Elevated circulating MDSCs in peripheral blood of BC patients was found directly correlated with cancer stage and metastasis [88, 89], but information on the role of MDSCs in human BC is limited and requires more studies.

#### 2.1.4. Mast Cells (MCs) and Natural Killer Cells (NKs)

Mast cells (MCs) are granulocyte-derived myeloid cells, containing histamine and heparin-rich granules, classically associated with allergic disorders. They display 2 phenotypes producing different mediators with opposite roles in tumorigenesis (antitumorigenic MC1 and protumorigenic MC2), depending on the biochemical milieu of the TME and tumor cells themselves [90, 91]. MCs are recruited by several cancer cell-derived cytokines and chemokines (i.e., osteopontin, CXCL8, CCL2, CXCL1, and CXCL10) into TME and can directly interact with infiltrated immune cells, tumor cells, and ECM [90]. MC1 are cytotoxic producing granzyme B, IL-9, and histamine, which induces DC maturation and inhibits tumor growth in murine models. In contrast, MC2 produces a variety of angiogenic and metastatic substances, i.e., VEGFs, fibroblast growth factor (FGF), matrix metalloproteinase-9 (MMP-9), TGF-*β*, and cytokines (i.e., IL-1*β*, IL-6, and IL-13) [90].

Peripheral to mammary adenocarcinoma [92], MCs contribute to tumor invasiveness and metastatic spread through the secretion of MMPs and tryptase which promote ECM disruption [91, 93–96] and differentiation of myofibroblast [97, 98]. Additionally, MCs contribute to neovascularization by releasing classical (i.e., VEGF, FGF-2, platelet-derived growth factor (PDGF), and IL-6) and nonclassical proangiogenic factors (mainly tryptase and chymase) [99]. Tryptase and chymase are suggested to be involved in cancer progression [100]. However, the infiltration of chymase-positive and tryptase-positive MCs in BC tissues is significantly higher in luminal A and luminal B subtypes compared to TNBC and HER2^+^ subtypes [101]. This study showed that higher MC numbers are associated with a less aggressive cancer type, suggesting that these MCs relate with more favorable cancer immunophenotype and might be beneficial prognostic indicators. The controversial findings may be due to the variation in the subpopulation of MCs and location of the cells in cancer tissue.

Natural killer cells (NKs) are a major antitumor component of the innate immune response. Similar to CTLs, NK-mediated tumor cell cytotoxicity depends on stimulatory and inhibitory signaling balance, such as cytokines (i.e., IFN and IL-2), affecting activating (i.e., NKG2D and CD161), inhibitory NK receptors (i.e., CD158a and CD158b), and signaling molecules [102]. As demonstrated in chronic viral infection, autoimmunity, and transplantation, NKs limit T cell function by cytokines, interactions with NKG2D and NKp46 receptors, or perforin-mediated T cell death [103]. In BC, NKs could enhance the activity of HER2 therapeutic antibodies by coupling NK cell antitumor function with immune checkpoint blocking, stimulatory antibodies, cytokines, or toll-like receptor (TLR) agonists [104]. The expression of a high proportion of ligands for NK-activating receptors positively correlates with survival indicators; however, a restricted number of ligands associate with worse prognosis, becoming potential biomarkers of BC progression [105].

### 2.2. Tumor Microenvironment and CAFs in BC

CAFs are derived from normal resident fibroblasts, cancer cells, adipocytes, and endothelial cells [18] with cancer stem cells differentiated into BC CAFs, via a paracrine effect of cancer cell-derived osteopontin [106]. CAFs are critical cells in metabolic TME reprogramming and therapy resistance in BC as they promote tumor proliferation, invasion, and metastasis [10–13].

#### 2.2.1. CAF Markers and Their Important Impact in BC Progression

Breast CAFs differentially express genes compared to normal fibroblasts, accounting for BC occurrence and progression [107], thus becoming predictive CAF molecular markers in tumor progression. In BC, CAFs are heterogeneous and divide into 4 population groups: CAF-S1 to CAF-S4, according to differential activation marker expression mainly including *α*-smooth muscle actin (ASMA), fibroblast activation protein (FAP), PDGF receptor *β* (PDGFR*β*), fibroblast-specific protein-1 (FSP-1), caveolin-1 (CAV-1), and CD29, with all correlating with poor prognosis (Table 1). All CAF subsets have low CAV-1 levels. The CAF-S1 subset mostly expresses all 6 markers, with especially FAP and ASMA highly expressed; the CAF-S2 subset expresses low levels of the 6 markers; the CAF-S3 subset is ASMA- and FAP-negative, but positive for the remaining 4 markers; and the CAF-S4 subset is characterized with no FAP, but high ASMA and CD29 [108]. Regarding localization, CAF-S1 and CAF-S4 are mainly in TNBC tumors, with HER2^+^ tumors additionally presenting CAF-S4. CAF-S3 has juxtatumoral localization in HER2^+^ and TNBC tumors. Lastly, CAF-S2 is in both tumor and juxtatumor compartments, mainly in the luminal A subtype [108]. Meta-analysis showed that increased number of activated tumor-infiltrating fibroblasts significantly correlated with poor clinical outcome of BC patients [109]. Additionally, CAFs with high ASMA and low high-mobility group box 1 (HMGB1) expression in cancer cells predict OS of invasive ductal BC patients [110]. Therefore, tenascin (TNC) overexpression (an ECM glycoprotein) is a poor prognosis factor [111], with FSP-1^+^ or podoplanin^+^ CAFs also associated with poor OS in BC patients [109]. Furthermore, CD10^+^ and GPR77^+^ CAFs promote tumor formation and chemoresistance by providing a survival niche for cancer stem cells [112]. CD44 is another cancer stem cell marker in CAFs, related to cancer cell survival and drug resistance [113]. Finally, CAFs expressing PDGFR*β* associate to metastasis and reduced tamoxifen response [114]. The vimentin- (VIM-) positive fibroblasts showed spindle cells in cytology of BC tissues [115]. Additionally, glucocorticoid receptor (GR) was observed in most of the fibroblasts in BC, especially luminal A subtype [116]. Since TME participates in several processes of tumor progression, the CAF tumorigenic functions highlight the importance of their targeting as a useful strategy to overcome BC.

#### 2.2.2. Secreted Substances from CAFs in BC

**Figure 2:**
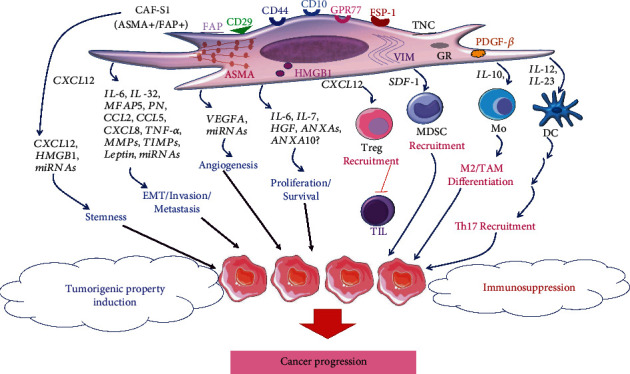
CAF markers, CAF-derived substances, and their tumor-promoting functions of aggressive CAFs in BC, CAF-S1, with positive stains of ASMA and FAP. CAFs can also produce a variety of cytokines/chemokines into TME, promoting cancer aggressiveness by direct effect on tumor cells to induce tumorigenic properties, i.e., stemness, EMT/invasion/metastasis, cell growth, and angiogenesis. Lastly, some substances can indirectly activate cancer progression through immune cells, thus modulating the immunosuppressive condition in BC tissues.

The most significant effect of CAFs is the secretion of tumor-promoting cytokines and chemokines, many of them playing a role in BC (Figure 2). One of them, CXCL12/SDF-1, shows strong expression in IL-7-producing fibroblasts, with the CXCL12/CXCR4 axis impacting tumor cell stemness promoting BC growth [117]. CAF-derived IL-6 and hepatocyte growth factor (HGF) induce androgenic enzymes contributing to intratumoral androgen metabolism in ER^−^ BC patients [118]. The matricellular protein periostin (PN), secreted mainly by CAFs, binds to cancer cell surface receptors activating progression in invasive ductal breast carcinoma (IDC), showing increased PN levels compared to ductal carcinoma *in situ* (DCIS) [119]. Interestingly, high CAF-derived PN levels in IDC correlate with increased tumor malignancy grade and shorter patient OS, suggesting PN participation in IDC progression [119]. CAF-secreted microfibrillar-associated protein 5 (MFAP5) promotes cancer cell EMT marker upregulation, migration, and invasion via the Notch1 pathway [120, 121]. Prometastatic chemokines CXCL8 (IL-8), CCL2 (monocyte chemoattractant protein-1 (MCP-1)), and CCL5 (RANTES) are upregulated in TNBC cell lines cocultured with primary CAFs from BC patients, acquiring invasive properties mediated by tumor necrosis factor-alpha- (TNF-*α*-) induced Notch1 activation [122, 123]. Moreover, MMPs expressed by the BC stroma correlate with metastasis [124], such as CAF-derived MMP-9, MMP-11, and tissue inhibitor of metalloproteinases-2 (TIMP-2) associating with poor prognosis in luminal A tumors [125].

Elevated CXCL1 in BC stroma correlates with increased tumor grade, disease recurrence, and poor patient survival [126] and inversely correlates with TGF-*β* signaling component expression. Additionally, CAF-inhibited TGF-*β* signaling *in vitro* increases CXCL1 expression, suggesting a contribution in BC progression [126]. Another CAF-derived cytokine promoting BC cell invasion is IL-32, binding to cell membrane integrin *β*3 to activate downstream p38 MAPK signal transduction [127]. The adipocyte-derived cytokine, leptin, influences BC cell proliferation [128] enhancing ER signaling and mediated tumor-stroma interaction by short autocrine loop. Finally, CAFs secrete leptin and express its receptor, enhancing BC cell motility and invasiveness [129].

Autophagy, a self-degradative process, regulates tissue homeostasis and intracellular energy source and participates in tumor recurrence/prognosis [130]. Autophagic CAFs, with increased LC3II (autophagosome protein) expression, release HMGB1 activating TLR4 in luminal BC cells, enhancing stemness and tumorigenicity, and predicting increased relapse rate and poor prognosis [131]. In TNBC, CAF autophagy enhances cell proliferation and the EMT process (migration, invasion), through the Wnt/*β*-catenin pathway [132].

**Figure 3:**
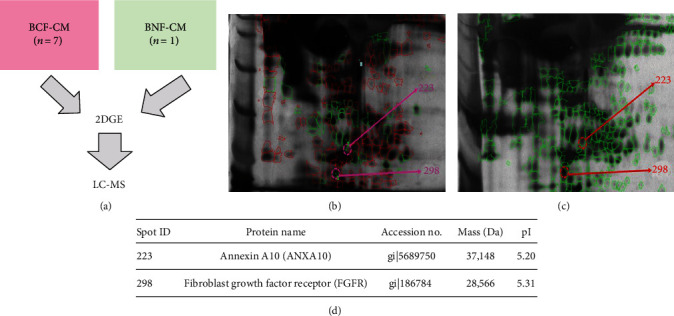
Two-dimensional gel electrophoresis (2DGE) and liquid chromatography mass spectrometry (LC-MS) of breast CAF conditioned-medium (BCF-CM). (a) The primary culture fibroblasts including 7 BCF-CM and 1 breast normal fibroblast-conditioned medium (BNF-CM). 100 *μ*g of each CM was prepared and applied to a 7 cm linear pH3-10, immobilized pH gradient (IPG) (GE Healthcare) strip. The first dimensional separation was performed at 20°C for 14 h in 3 steps including (1) 500 V for 1 h, (2) 1,000 V for 1 h, and (3) 5,000 V for 2-3 h. The second dimensional separation was performed by 12.5% SDS-PAGE at 150 V for 2 h. Gels were stained with silver (PlusOne staining kit, GE Healthcare) for 5 h. The gels were scanned by a Typhoon Laser Scanner (GE Healthcare). Protein spots were detected and percentages of the spot volume were calculated with Image Master 2D platinum program. (b, c) The representative gels of BNF-CM and BCF-CM, respectively. Spots no. 223 and 298 are found in only BCF-CM but not in BNF-CM. (d) Potential cancer-associated substances from breast CAFs determined by LC-MS. The protein spots in the gels from (b) and (c) were digested with trypsin. 100 *μ*l of 3% H_2_O_2_ was added, removed, and dehydrated with 100 *μ*l of 100% acetonitrile (ACN). Reduction was performed by adding 10 mM DTT in 10 mM NH_4_HCO_3_ and incubated at 56°C for 1 h. The solution was removed and replaced by adding 100 mM iodoacetamide in 10 mM NH_4_HCO_3_ and then dehydrated with 100% ACN. Digestion was performed by incubating with 20 ng/*μ*l trypsin (Promega Corporation). 10 mM NH_4_HCO_3_ was added on gel pieces and incubated at 37°C overnight. The proteins were extracted from gels by adding 0.1% formic acid (FA) in 50% CAN and resuspended with 0.1% FA in LC-MS water before injection into the LC-MS (SYNAPT™ HDMS Mass Spectrometer, Water Corp, UK) (lab data).

All the above has led our group to study breast CAFs isolated from fresh cancer tissues [133] and their secreted substances. Utilizing two-dimensional gel electrophoresis (2DGE), followed by liquid chromatography mass spectrometry (LC-MS), we found annexin A10 (ANXA10) and FGF receptor (FGFR) upregulation (Figure 3). Accordingly, ANXA are a family of closely related calcium- and membrane-binding proteins playing important roles in calcium signaling, cell division, apoptosis, and cell differentiation [134]. In prostate cancer, ANXA1 stimulates cell proliferation and dedifferentiation of cancer cells to stem-like cells [135]. ANXA3 is overexpressed in lung cancer CAFs, playing an important role in chemoresistance [136]. By interacting with cancer cells, extracellular CAF vesicles (containing ANXA6) induce aggressive pancreatic ductal carcinoma [137]. Additionally, ANXA10 correlates with the progression of pancreatic early lesions towards ductal adenocarcinoma [138]. In serous epithelial ovarian cancer, high ANXA10 level was found and correlated with chemotherapeutic response [139]. It has also been proposed as the poor prognostic marker in several cancers, i.e., serous epithelial ovarian carcinoma, papillary thyroid cancer, and perihilar and distal cholangiocarcinoma [139–141]. Though no information is available on ANXA10 in BC to date, it possibly plays critical roles in disease aggressiveness.

Upregulation of FGFR can undergo shedding from CAFs, although FGF5 (a receptor ligand) is released from CAFs inducing FGFR2 expression in HER2^+^ BC cells resulting in HER targeted therapy resistance [142]. FGFR1 amplification occurs most frequently in patients with luminal B-like BC and appears to correlate with patient's poor prognosis, although with no statistical significance [143]. FGF overproduction may autocrinally activate FGFR expression rendering its shedding out into the TME; thus, the FGF/FGFR axis in BC offers target molecules to attenuate cancer progression [144]. Potentially, the combinations of anti-FGFR or anti-FGF therapies with checkpoint inhibitors can improve survival and quality of life of BC patients with novel and increasingly accurate therapeutic strategies.

Studies reveal a paracrine effect of CAF secreted factors over tumor cells enhancing therapeutic resistance through the evasion of apoptotic cell death [145]. HMGB1, a CAF-mediated protein, induces doxorubicin (a chemotherapeutic used to treat BC) resistance through autophagy induction [133]. Moreover, CAF-derived IL-6 secretion induces resistance to trastuzumab (a monoclonal antibody against HER2) by cancer stem cell expansion and apoptosis reduction via NF-*κ*B, JAK/STAT3, and PI3K/AKT signaling pathways [146]. Therefore, a novel strategy reverting trastuzumab resistance in HER2^+^ BC could be the combination of an anti-IL-6 antibody with these specific pathway inhibitors [146]. When cancer cells are cocultured with CAFs, tumor cells show chemoinsensitivity typical of aggressive BC [147], partly due to chemotherapy-induced metabolic and phenotype transformation of healthy fibroblasts into CAFs. This generates a nutrient and inflammatory cytokine-enriched environment, activating stemness in adjacent BC cells [148]. A combined drug approach, bortezomib (a proteasome inhibitor) and panobinostat (a histone deacetylase inhibitor), synergistically decreases patient-derived CAF viability by inducing caspase-3-mediated apoptosis [149].

Other important mediators of intracellular communication currently emerging are exosomes, which affect BC progression by horizontally transferring microRNAs (miRs), mRNAs, and proteins [150]. Differential expression profile analysis identified three miRs (miR-21, miR-378e, and miR-143) increased in exosomes from breast CAFs compared to normal fibroblast, proposing their role in stemness and EMT induction in BC cell lines [151]. Breast stromal fibroblasts can secrete the proangiogenesis protein vascular endothelial growth factor A (VEGF-A) which can be repressed by p16(INK4A) [152]. The above evidence supports the potential therapeutic strategies targeting breast CAFs, offering new tools in BC therapy [153, 154].

## 3. CAF Immunosuppressive Effect in BC

Immune modulation in breast TME plays a central role in determining disease outcome and immunotherapy response, in particular immune checkpoint modulators [155]. Accordingly, CAFs are key modulators of TME and immune response, secreting soluble molecules, such as cytokines/chemokines (refer to Table 1, Figure 2). This process allows CAFs to create immunological barriers against CD8^+^ T cell-mediated antitumor immune responses [39]. Targeting CAFs with an on-shelf antifibrotic agent, TGF-*β* antagonist, combined with doxorubicin, inhibits tumor growth and metastasis synergistically [156]. Additionally, elimination of FAP^+^ CAFs *in vivo* shifted the immune microenvironment from Th2 to Th1 polarization, suggesting CAFs as attractive targets in metastatic BC [157]. Several clinical trials are ongoing concerning the role of the immune system in BC editing, with potential impact of immunotherapy [36]. Specifically, the CAF-S1 subtype in BC increases recruitment and differentiation of CD4^+^CD25^+^ Tregs in TME, through CXCL12/SDF1-*α* leading to the inhibition of effector T cell function [108].

Alternatively, CAF activation positively correlates with increased CD163^+^ TAM infiltration and lymph node metastasis in TNBC patients [158], being prognostic factors for disease-free survival.

An interesting epigenetic mediator is fibrotic histone deacetylase 6 (HDAC6), programming an immunosuppressive TME that reduces antitumor immunity, possibly a good target enhancing BC immunotherapy [159]. Genetic or pharmacologic disruption of HDAC6 in CAFs delays tumor growth, inhibits tumor recruitment of MDSCs and Tregs, alters macrophage phenotype switch, and increases CD8^+^ and CD4^+^-T cell activation *in vivo* [159]. Prostaglandin E2/cyclooxygenase-2 (COX2) is the main target of HDAC6 in CAFs, and COX2 overexpression in HDAC6-knockdown CAFs reinstates fibroblast immunosuppressive properties.

Finally, TME-secreted substances also affect DC function in some cancers [160]. Accordingly, cervical cancer cells regulate DC production of IL-23 and IL-12 in DC/fibroblast cocultures through IL6/C/EBP*β*/IL1*β* promoting Th17 cell expansion, although reducing antitumor Th1 differentiation during cancer progression [160].

## 4. Conclusion and Future Perspectives

A successful cancer therapeutic strategy using the immune system as a target requires understanding of cellular components in TME. Characteristics of CAFs and their secreted substances in each BC type are not only useful for being targets of treatment but also prognostic and predictive factors. According to secretory substances, cytokines/chemokines, together with exosomes containing miRs, mRNAs, and proteins, CAFs become key modulators for cancer progression and immune cell polarization, resulting in protumoral or immunosuppressive status in the TME. The use of appropriate biomarkers, resulting from cancer cell-immune cell-CAF crosstalk, will identify promising cases responding to therapy, enabling a suitable therapeutic strategy. Lastly, understanding the molecular mechanisms of complex interactions between CAFs and immune cells is needed to fill the knowledge gap, thus providing potential targets for improved cancer therapy.

## Figures and Tables

**(a):** 

Category	Markers (Ref)
Characteristic of CAFs	ASMA/CAV-1/CD29/FAP/FSP-1/PDGFR*β* [108], VIM [115], CD10 [112]
Poor prognostic CAFs	ASMA/HMGB1 [110], COX-2 [159], CXCL-1 [126], FSP-1/podoplanin [109], HDAC6 [159], LC3/Snail1/TLR-4 [131], TNC [111], VIM [115]
Chemoresistance induction CAFs	CD10 [112], CD44 [113], GPR77 [112], IL-6 [118], PDGFR*β* [114], HMGB1 [133], IL-7 [117]
Immune cell suppression CAFs	IL-10 [40, 43], IL-12/IL-23 [160], Chi3L1 [161], CXCL12/SDF-1 [108], CXCL16 [162]

**(b):** 

CAF-derived substances	Target cells	Effects	Ref
CXCL1	Cancer cells	Decrease TGF-*β* signaling, promote tumor progression	[126]
CXCL8/IL-8	Cancer cells	Mediate the prometastatic activities	[123]
CXCL12/SDF-1*α*	CD4^+^CD25^+^ Tregs	Attract, increase survival and promote differentiation to a regulatory phenotype	[108]
	MDSCs	Recruit and exert tumor-promoting effects	[163]
CXCL16	Monocytes	Promote stroma activation in TNBC	[162]
Chi3L1	CD8^+^CD4^+^ T lymphocytes	Enhance tumor infiltration and promote Th1 phenotype	[161]
	Macrophages	Recruit and differentiate into M2-like phenotype	[161]
TNF-*α*, IL-1*β*, IL-6, and IL-12p70	T lymphocytes	Secrete IFN-*α* and IFN-*γ*, induce CTL responses	[164]
IL-6	Cancer cells	Induce EMT and promote tumor progression	[165]
IL-32	Cancer cells	Promote cancer cell invasion	[127]
Leptin	Cancer cells	Promote cancer growth and progression Enhance cancer cell motility and invasiveness	[128, 129]
MCP-1, SDF-1	Macrophages	Recruit and differentiate into M2-like phenotype	[166]

## Data Availability

Data availability is upon request to the corresponding author.

## Abbreviations

ASMA: *α*-Smooth muscle actin
M2: Alternatively activated macrophages
APCs: Antigen-presenting cells
BC: Breast cancer
CAFs: Cancer-associated fibroblasts
CAV-1: Caveolin-1
Chi3L1: Chitinase 3-like 1
M1: Classically activated macrophages
COX: Cyclooxygenase
CTLs: Cytotoxic T cells
CTLA-4: Cytotoxic T lymphocyte-associated antigen 4
DCs: Dendritic cells
DCIS: Ductal carcinoma *in situ*
HER2: Epidermal growth factor receptor
EMT: Epithelial-mesenchymal transition
ER: Estrogen receptor
ECM: Extracellular matrix
FAP: Fibroblast activation protein
FGF: Fibroblast growth factor
FSP-1: Fibroblast-specific protein-1
FOXP3: Forkhead box P3
Th17: Helper T17
HGF: Hepatocyte growth factor
HMGB1: High-mobility group box 1
HDAC6: Histone deacetylase 6
HER-2: Human epithelial growth factor receptor-2
HLA: Human leukocyte antigen
IFN: Interferon
IL: Interleukin
DCIS: Invasive ductal breast carcinoma
LC-MS: Liquid chromatography mass spectrometry
MHC: Major histocompatibility complex
MMPs: Matrix metalloproteinases
MCs: Mast cells
MFAP5: Microfibrillar-associated protein 5
MCP-1: Monocyte chemoattractant protein-1
MICs: Monocyte-infiltrating cells
MDSCs: Myeloid-derived suppressor cells
NKs: Natural killer cells
OS: Overall survival
PN: Periostin
PDGF: Platelet-derived growth factor
PDGFR*β*: Platelet-derived growth factor receptor-*β*
PR: Progesterone receptor
PD1: Programmed death 1
PD-L1: Programmed death-ligand 1
Tregs: Regulatory T cells
RFS: Relapse-free survival
SDF-1: Stromal cell-derived factor 1
TGF-*β*1: Transforming growth factor-*β*1
TNBC: Triple-negative breast cancer
TAMs: Tumor-associated macrophages
TILs: Tumor-infiltrating lymphocytes
TME: Tumor microenvironment
TNF-*α*: Tumor necrosis factor-*α*
TNC: Tenascin C
TIMP-2: Tissue inhibitor of metalloproteinases-2
TLR: Toll-like receptor
TGF-*β*1: Transforming growth factor-*β*1
TAP: Transporter-associated antigen processing
2DGE: Two-dimensional gel electrophoresis
VEGF: Vascular endothelial growth factor.
